# Dr. Wardlaw

**Published:** 1893-12

**Authors:** 


### Obituary.

IN MEMORY OF DR. WARDLAW.—A meeting of the Atlanta dentists was held in the Kimball House reading room Thursday morning, September 4, to take action on the recent death of Dr. W. C. Wardlaw.

Dr. B. H. Catching was elected chairman and Dr. M. Z. Crist, secretary. Dr. Catching made a short talk, in which he paid a high tribute to the character of Dr. Wardlaw. Speeches of the same tenor were made by Drs. Browne, Holland, Rosser, Crenshaw, Mr. Selby and others.

A committee of five, consisting of Drs. Catching, Crenshaw, Holland, Browne and Rosser, reported the following resolutions, which were adopted and their publication requested in the daily press of Atlanta and Augusta, *The*

*Southern Dental Journal and Luminary* and *The Dental Cosmos :*

"We have assembled to pay tribute to the memory of our esteemed and beloved brother practitioner, the late Dr. W. C. Wardlaw. Only recently we were rejoicing that he had selected our city as his home. We rejoiced that in his citizenship we were to have a man of spotless character and the purest integrity—one who loved God and his fellow men.

"We rejoiced that there had been added to our local ranks a man of eminent attainments in literature, art and science; one whose reputation was not confined to a continent; one whose innate modesty shrank from publicity, and whose sympathies extended to and aided the most humble; one who was by nature a gentleman, by profession a Christian, by attainments a leader.

"But, alas! He had only reached our gates and entered our walls when death called him to his eternal home.

"To say that we realize our loss does not express our feelings; yet we feel that such a life, crowned with such attainments, is not lost, but shall live with us as a guide and pattern, which will, if we follow it, tend to our perfection morally, socially and professionally.

"May we emulate his many virtues and strive after his attainments.

"Realizing the power and wisdom of God, we humly bow to his will.

"Our deepest sympathies we extend to his bereaved wife, children and relatives. Their loss is our loss, but his eternal gain.

"B. H. CATCHING,
"WM. CRENSHAW,
"S. G. HOLLAND,
"C. V. ROSSER,
"W. G. BROWNE,
"*Committee.*"

—*Southern Dental Journal.*

